# Hypoxia and viral infectious diseases

**DOI:** 10.1172/jci.insight.147190

**Published:** 2021-04-08

**Authors:** Richard Huang, Melissa Huestis, Esther Shuyi Gan, Eng Eong Ooi, Michael Ohh

**Affiliations:** 1 Department of Laboratory Medicine and Pathobiology, University of Toronto, Toronto, Ontario, Canada.; 2 Programme in Emerging Infectious Diseases, Duke-NUS Medical School, Singapore, Singapore.; 3 Department of Microbiology and Immunology, National University of Singapore, Singapore, Singapore.; 4 Saw Swee Hock School of Public Health, National University of Singapore, Singapore, Singapore.; 5 Department of Biochemistry, University of Toronto, Toronto, Ontario, Canada.

## Abstract

Oxygen-sensing mechanisms allow cells to adapt and respond to changes in cellular oxygen tension, including hypoxic conditions. Hypoxia-inducible factor (HIF) is a central mediator in this fundamental adaptive response, and has critical functions in normal and disease physiology. Viruses have been shown to manipulate HIFs during their life cycle to facilitate replication and invasion. Conversely, HIFs are also implicated in the development of the host immune system and response to viral infections. Here, we highlight the recent revelations of host-pathogen interactions that involve the hypoxic response pathway and the role of HIF in emerging viral infectious diseases, as well as discussing potential antiviral therapeutic strategies targeting the HIF signaling axis.

## Introduction

**Figure 1:**
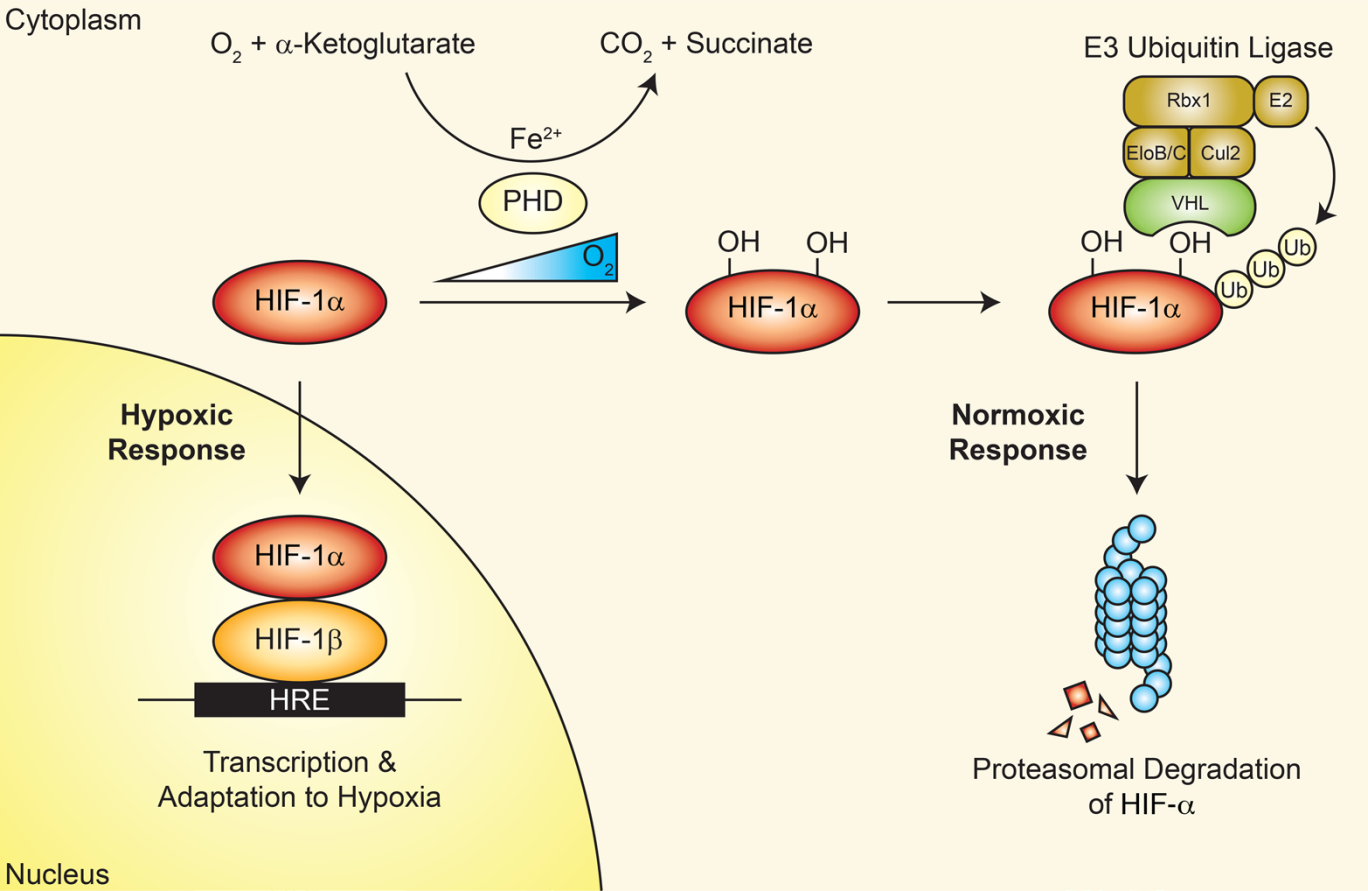
The canonical oxygen-sensing pathway involving HIF. HIF-1α is regulated through an oxygen-dependent mechanism that involves the hydroxylation of proline residues by PHDs and essential cofactors (including α-ketoglutarate and iron). Afterward, the E3 ubiquitin ligase complex (comprising VHL, Elongin B/C [EloB/C], Cul2, Rbx1, and E2) ubiquitinates HIF-1α, which results in its proteasomal degradation. However, under hypoxic conditions, PHDs are unable to perform their function, and HIF-1α is able to associate with HIF-1β within the nucleus to initiate the transcriptional responses for hypoxic adaptation.

The cellular environment is in constant flux. To sustain homeostasis, cells must be able to readily adapt to perturbations in various conditions, such as oxygen levels. When oxygen tension decreases, cells experience stress and respond accordingly to facilitate survival. Regulation of this stress response is governed by hypoxia-inducible factor (HIF). HIF is a transcription factor that is activated by hypoxia and in turn triggers a response to hypoxia by promoting the transcription of numerous genes that encode proteins for appropriate adaptation to compromised oxygen availability. Under normoxia, the HIF-1α isoform undergoes proteasomal degradation following hydroxylation on 2 proline residues by oxygen-dependent prolyl hydroxylases (PHDs), and subsequent ubiquitination by the von Hippel–Lindau–containing (VHL-containing) E3 ubiquitin ligase complex. Conversely, PHDs are not active in the absence of oxygen; therefore, proline hydroxylation of HIF is abrogated under hypoxia, allowing it to escape degradation and accumulate within the nucleus to promote the transcription of hypoxia-responsive genes (Figure 1) (1, 2). The discovery of VHL, a tumor suppressor, was the result of the search for the gene driving the predisposition to and development of VHL disease. An individual born with one defective *VHL* allele develops VHL disease upon loss of the remaining WT allele, with the disease characterized by visceral cysts along with benign and malignant tumors in multiple organs including the kidney (3). Notably, the discoveries that directly linked VHL to HIF led to the seminal elucidation of the canonical oxygen-sensing pathway (4–8). Although HIF was originally discovered as a mediator of oxygen sensing, its role in development has since expanded to areas of research such as viral pathogenesis and immunity (9, 10).

While oxygen is required for the survival of all human cells, not all organs receive the same amount of oxygen (11). Between respired air and the cytoplasm of cells, there exists an oxygen gradient that drives the diffusion of oxygen into peripheral tissues. Termed the “oxygen cascade,” this gradient illustrates how oxygen levels in most organs, with a few exceptions, are generally lower than that of atmospheric air (12). The tissue oxygen microenvironments, in which host cells reside, are essential factors that are often overlooked during in vitro studies of viral pathogenesis. As a result, in vitro experiments, which are mostly performed under normoxic conditions, may not recapitulate disease phenotypes that develop in hypoxic microenvironments. Viral infection is influenced by multiple factors in host cells, such as differential gene expression, pathway activation, metabolic states, and receptor expression — all of which can be influenced by oxygen availability (9). Likewise, oxygen levels also affect the innate and adaptive immune response to infection.

## Hypoxia and the immune response to viral infections

**Figure 2:**
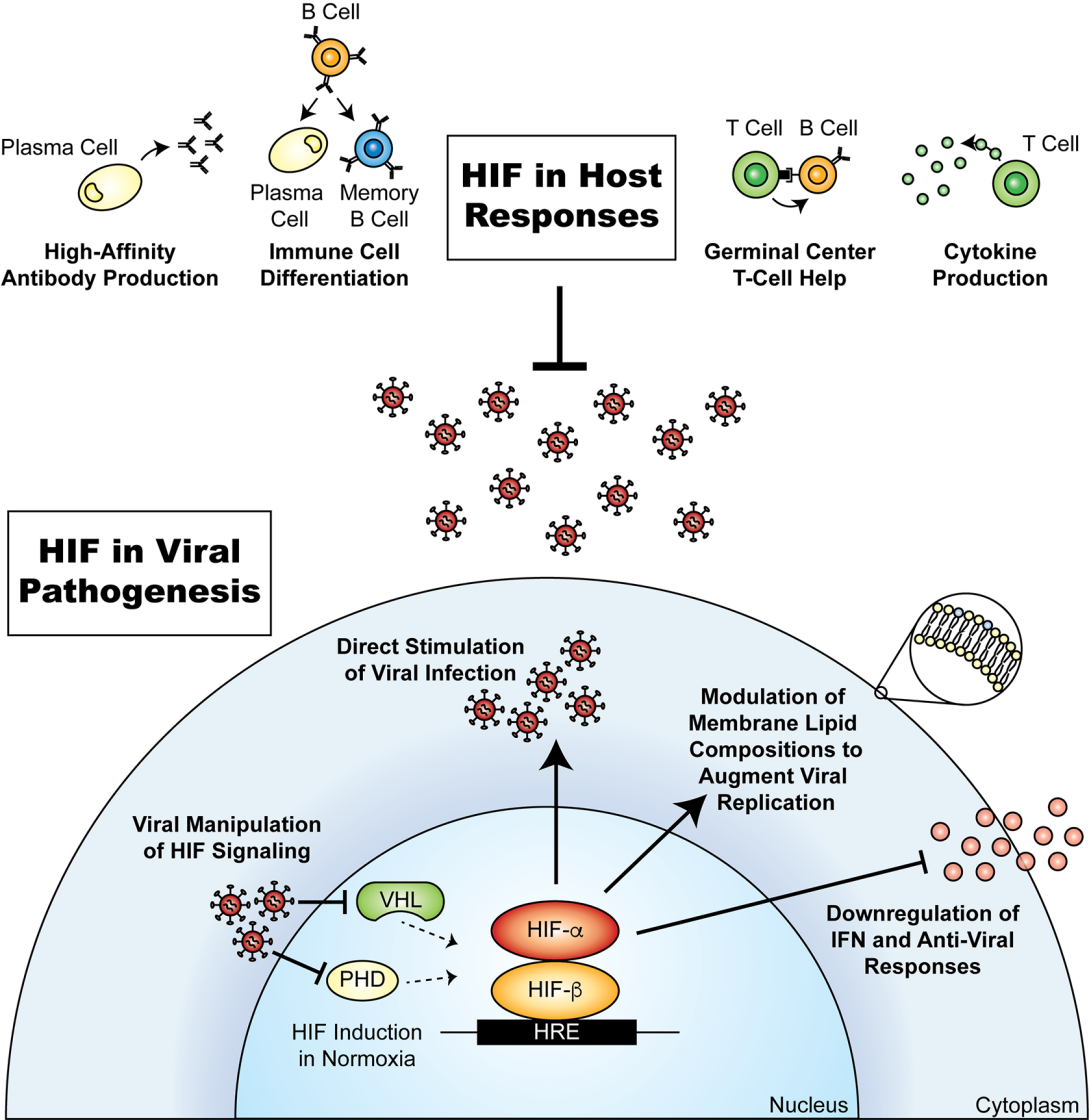
The involvement of HIF in viral pathogenesis and host responses. HIF can be involved in various viral mechanisms of infection and in the host antiviral response. Certain viruses can manipulate the HIF pathway by inhibiting the degradation of HIF-α that leads to HIF stabilization, which may result in cellular responses to increase viral replication and downregulate antiviral responses. Conversely, HIF plays a central role in immune responses against viruses by contributing to the development of B cells in germinal centers through T cell help, regulation of immune cell differentiation, and production of high-affinity antibodies. These actions of HIF reveal its duality in participating in both an antiviral and a propathogenic response.

The host response against viruses involves the coordination of the innate and adaptive immune systems, which can recognize various components of the virus and mount a response to clear the infection. Studies in the past 2 decades have recognized that hypoxia and HIF regulate immune cells by influencing their metabolism, function, differentiation, and survival, which has implications for immune responses and development (Figure 2) (10). Furthermore, areas of immune development and homeostasis, such as the BM, lymphoid organs, and germinal centers, have differing oxygen tensions (13). HIF thus plays a major role in shaping the immune response to viral infection.

The contribution of hypoxia and HIF to innate immune responses has previously been reviewed in detail (10, 14). HIF-1α is shown to support innate responses and the initiation of adaptive immunity through actions on myeloid cells, cytokine expression, and antigen presentation, as well as being implicated in NF-κB signaling, which is a critical regulator of innate immunity (10, 15).

Besides the innate immune response, HIF has also been implicated in the function of both T and B lymphocytes, which are principal components of the adaptive immune system and thus response to viral infections (Figure 2) (10, 13, 16). The humoral response involves the development of antiviral antibodies in B lymphocytes that play an essential role in the prevention of pathogen dissemination and elimination of viral infections. Immunity is also sustained through the development of long-lived plasma cells and memory B lymphocytes (Figure 2) (17). Development of such B lymphocyte responses occurs in the germinal centers of lymph nodes and the spleen through the function of T follicular helper (Tfh) lymphocytes. Recent reports have revealed a crucial role for VHL and HIF in regulating the function of Tfh cells to promote (i) B cell differentiation into antibody-producing plasma cells and memory B cells following infection and immunization, and (ii) the production of high-affinity antibodies (17, 18). These responses are required in order to increase the specificity and efficacy of the host immune response (18). Using conditional knockouts in T lymphocytes and mouse models of infection, VHL was determined to negatively regulate a HIF-mediated shift toward glycolytic metabolism — a key metabolic event for the initiation of Tfh cell development (18). Lack of VHL results in deficiencies in the development of Tfh cells and the functions of germinal centers, which impacts the quality of the humoral response (18).

In addition to Tfh cells, low-oxygen environments have been shown to impact the activity of T cells. While cytotoxic T cells proliferate at a slower rate under hypoxic conditions, they have higher concentrations of granzyme B within granules, in turn leading to more efficient killing of target cells (19, 20). It has also been previously observed that CD4^+^ T cells stimulated under hypoxic conditions secrete higher levels of IL-4 and IFN-γ, both effector cytokines that regulate the host immune response (21). As both the innate and adaptive immune responses are key host responses to viral infection, oxygen tension thus plays an important role in regulating the duration and perhaps even persistence of viral infection.

## Viral invasion strategies in hypoxia

In addition to regulating the immune response, oxygen tension plays a direct role in shaping viral pathogenesis. Several viruses have evolved strategies to take advantage of hypoxic conditions to infect areas of the body that have a lower oxygen tension than respired air (22). Indeed, cellular adaptations to hypoxia have been shown to be directly or indirectly advantageous to several viruses, such as dengue virus (DENV), HCV, Kaposi sarcoma–associated herpesvirus (KSHV), and EBV (Table 1) (9, 23).

One of the most direct effects of HIF-1α on viral replication can be observed with KSHV. KSHV was the first virus to have a functional hypoxia-regulatory element (HRE) identified within its genome upstream of the *Rta* gene, which induces the lytic replication of the virus (24, 25). HRE-mediated regulation of *Rta* has been observed in primary effusion lymphoma cell lines to functionally trigger the lytic replication of KSHV under hypoxia (26). Similarly, HIF-1α activates lytic EBV infection by directly binding the promoter of the EBV latent-lytic switch, Zp, via an HRE that activates expression of its *BZLF1* gene, which promotes expression of early lytic genes (27). Therefore, hypoxia likely plays a prominent role in the regulation of gammaherpesviruses, including KSHV and EBV, as replication of these viruses is regulated by HIF-1α (Figure 2).

Hypoxia can also exert inhibitory effects on viral replication. For instance, replication of adenoviruses and HIV is known to be suppressed by hypoxia (28, 29). Under hypoxic conditions, HIV replication and transcriptional activity were significantly reduced compared with normoxic conditions. In specific strains of HIV that contain HREs in their long terminal repeat (LTR) regions, the interaction between HREs and the HIF-2α–HIF-1β complex results in inhibition of HIV replication (30). However, studies analyzing HRE-deficient HIV strains and in vivo experiments would be needed to further characterize the relevance of oxygen tension on HIV infection.

In addition to the dependence on direct HIF interactions, multiple viruses require activated HIF-1α to regulate various host cell processes for their benefit (Figure 2). For example, altered cellular protein or lipid metabolism due to exposure to hypoxia (31, 32) has been shown to augment DENV infection in myeloid cells (33, 34). At oxygen tensions similar to that within the lymph node (3% O_2_), which is a site of DENV infection (35), transcription and translation of FcγRIIA is upregulated by HIF-1α, resulting in increased uptake of the viral immune complex. However, this increase in receptor expression is not sufficient for enhanced infection. The elevated FcγRIIA expression is accompanied by an increase in ether-phosphatidylethanolamine (ether-PE) lipid compositions occurring independently of HIF-1α, resulting in significantly greater viral replication (33). Furthermore, DENV infection has been shown to manipulate cholesterol metabolism in hepatic cells cultured under conditions similar to that within the liver. DENV infection induces the secretion of proprotein convertase subtilisin/kexin type 9 (PCSK9), which reduces the activity of low-density lipoprotein receptor and further drives de novo cholesterol synthesis, resulting in a reduction in STING-induced type I IFN responses and downstream antiviral responses (Figure 2). Clinical data also showed a correlation between plasma PCSK9, increased viremia, and infection severity in affected patients (34).

Furthermore, even in the presence of oxygen, several viruses, such as HBV and vaccinia virus, have developed methods to interact with various elements of the HIF signaling pathway to promote infectivity. Virus-induced stabilization of HIF-1α stimulates the transcription of hypoxia-inducible genes. One such strategy is inhibition of HIF-1α interaction with PHDs and VHL. For example, HIF-1α stabilization is highly beneficial for the survival of HCV, as it enhances hepatic angiogenesis through the production and secretion of VEGF (36, 37). Similarly, vaccinia virus stabilizes HIF-1α by binding PHD2 directly and inhibiting its ability to target HIF-1α for degradation (36). This is particularly interesting, as the ability of certain viruses to adapt to hypoxic environments could shape the choice and development of oncolytic viruses (OVs) for cancer treatment (37). The rapid growth of malignant cells that often outstrips the rate of angiogenesis creates a hypoxic microenvironment in most tumor masses. OVs that mechanistically exploit hypoxia for infection and replication could be more effective in selectively targeting and destroying tumor cells while leaving noncancerous cells, which would be in higher-oxygen microenvironments, relatively unaffected. Moreover, the downregulation of cellular antiviral responses within the tumor as a trade-off for increased cancer growth could further augment cancer cells’ susceptibility to OV infections (38).

To date, viruses such as vaccinia virus, vesicular stomatitis virus (VSV), and adenoviruses have been developed as OV therapy platforms (39). These viruses have been selected due to the ease of cloning transgenes into their genomic backbone to generate OV candidates. However, not all of these viruses are able to take advantage of hypoxic microenvironments. Vaccinia virus can stabilize HIF-1α under normoxic conditions for its benefit (36), as previously mentioned, which suggests that its replication is enhanced in hypoxic environments. In contrast, VSV appears to be inhibited by increased HIF-2α signaling, which may explain why VSV is less potent than other viruses used in OV antitumor therapy (40). Similarly, adenovirus replication and its lytic activity are compromised under hypoxic conditions, which could limit the application of adenovirus-associated OVs as effective anticancer therapeutics (28, 41).

A deeper understanding of the virus-host interactions in low-oxygen environments could inform the choice and design of new, more effective OV therapies. Indeed, a study using genetically engineered herpesvirus to exploit the tumor hypoxic environment for enhanced oncolytic activity showed promising results in the treatment of colorectal metastases (42). Additionally, coadministration of HIF-α stabilizers or inhibitors with OV therapy could produce synergistic antitumor effects, depending on the specific type of OV treatment. However, further studies will be needed to understand how hypoxia can be exploited to improve the efficacy of OV therapies.

## The impact of hypoxia on respiratory viral infections

The human airway is rich in oxygen, as it is the site where oxygen is taken up by RBCs and distributed to distal organs and tissues. Nonetheless, respiratory viruses are able to take advantage of the HIF-1α signaling pathway. Indeed, studies have shown that HIF-1α may be stabilized by respiratory viruses, such as influenza and respiratory syncytial viruses (RSVs) (43, 44). Influenza A H1N1 infection was shown to stabilize HIF-1α through its inhibitory effects on proteasome-mediated HIF-1α degradation. Consequent nuclear accumulation of HIF-1α resulted in proinflammatory cytokine expression and secretion, causing severe inflammation (43). Similarly, RSV infection in pulmonary epithelial cells revealed oxygen-independent stabilization of HIF-1α protein and subsequent transcription of HIF-1α target genes that established an intracellular environment favorable for RSV replication (44).

Hypoxia could also play an important role in the pathogenesis of COVID-19, the etiological agent of which is severe acute respiratory syndrome coronavirus 2 (SARS-CoV-2). Similar to other betacoronaviruses of zoonotic origin — SARS-CoV and Middle East respiratory syndrome coronavirus (MERS-CoV) — SARS-CoV-2 can, in certain at-risk individuals, lead to the development of acute respiratory distress syndrome (ARDS), respiratory failure, and death (45–47).

SARS-CoV and SARS-CoV-2 bind to the same cellular membrane receptor, angiotensin-converting enzyme 2 (ACE2), for entry into host cells (47). ACE2 is a transmembrane glycoprotein that modulates inflammation and vasculature through regulation of the renin-angiotensin system (RAS) (47, 48). There is emerging evidence that ACE2 is regulated by HIF-1α and that oxygen tension may influence susceptibility to COVID-19 (49, 50). A recent study analyzing the degree of COVID-19 severity in infected individuals residing in high-altitude areas suggested that those with SARS-CoV-2 infection who had acclimated to hypoxic environments were less likely to have severe disease outcomes (51). The authors attributed the finding to hypoxia-induced downregulation of ACE2 expression in pulmonary artery smooth muscle cells occurring indirectly through HIF-1α activity (50, 51). Furthermore, analysis of the SARS-CoV-2 host-protein interactome identified numerous interacting proteins, such as those in the NF-κB and mTOR signaling pathways, as well as components of the Cullin 2–RING (CUL2–RING) E3 ligase complex; all of these components are part of the HIF-1α signaling pathway, which drives HIF-1α expression (46). Thus, SARS-CoV-2 infection could inhibit HIF-1α and its downstream activity. As HIF-1α influences multiple aspects of the immune system, this has potential implications for the immune system response to WT SARS-CoV-2 infection. Perhaps such interactions between SARS-CoV-2 and the HIF-1α signaling pathway underpin the slow clearance of SARS-CoV-2 RNA by the immune system. These immune system effects may also underpin susceptibility to SARS-CoV-2 reinfection. More work, however, is needed to establish the role the HIF-1α signaling pathway plays in these clinically observed responses.

## Therapeutic antiviral strategies targeting HIF signaling

**Figure 3:**
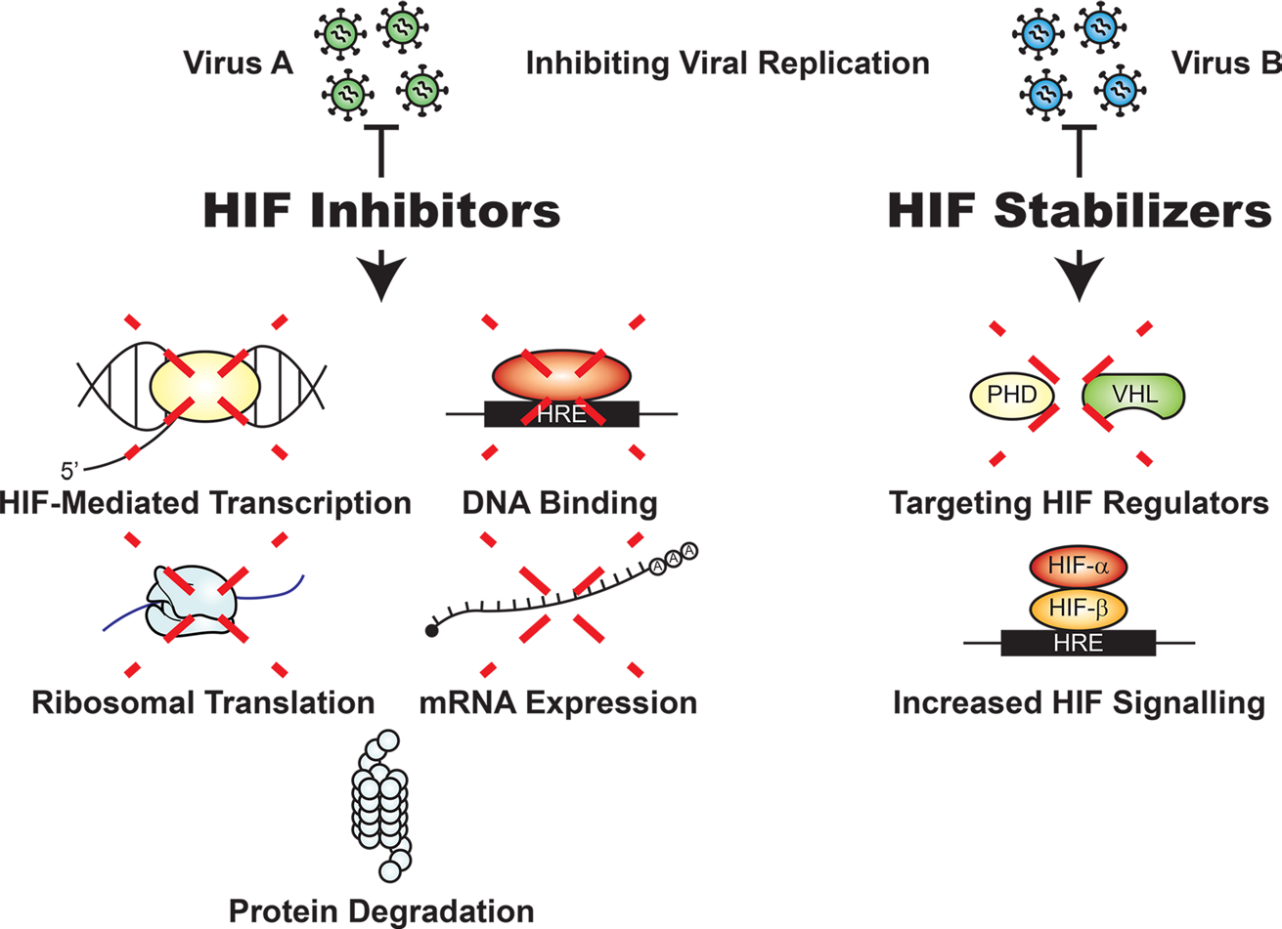
Therapeutic methods targeting HIF to inhibit viral replication. HIF stabilizers and inhibitors can act on various pathways involved in HIF signaling — such as HIF-α stabilization, ribosomal translation, mRNA expression levels, protein degradation, transcription, and binding to HREs — in order to disrupt the viral replicative life cycle. These different strategies depend on the role of HIF in either disrupting or assisting in infection and replication, which is dependent on the type of virus. Viruses such as HCV can manipulate the HIF response, whereas others, such as VSV, are inhibited. This highlights the need for further investigation in order to determine the role of HIF in facilitating or inhibiting infection and its therapeutic potential.

The process of developing effective drugs or vaccines to target viruses is notably challenging and complex. Currently, no broad-spectrum antiviral therapeutic exists, as drugs must be meticulously designed for each viral strain (52). However, despite their role in viral pathogenesis, no HIF-1α or oxygen-sensing pathway inhibitors have been investigated as potential antiviral therapies against a range of viruses. Currently, several HIF-1α and HIF-2α inhibitors have been developed or are in development as therapeutics for different forms of cancer (9, 53). Some of these therapeutics remain in clinical trials and target both HIF-1α and HIF-2α (53); however, most research remains focused on the role of HIF-1α in host-pathogen interactions. As a result, this discussion focuses on HIF-1α inhibitors, which are categorized according to 5 different mechanisms for modulating HIF-1α, targeting its (a) mRNA expression, (b) protein translation, (c) degradation, (d) DNA binding, and (e) transcriptional activity (Figure 3) (54).

Small molecule inhibitors such as the antisense oligonucleotide EZN-2968 that target HIF-1α mRNA expression are ideal (55), as protein translation is a limiting factor for HIF-1α expression in response to hypoxia. In addition, receptor tyrosine kinases and topoisomerases can also be used as targets to limit HIF-1α activity due, at least in part, to the crosstalk between these signaling pathways (56). Inhibitors of receptor tyrosine kinases, such as gefitinib, reduce the translation of HIF-1α, which appears to be mediated by the downstream PI3K/Akt pathway, as its inhibition also results in a reduction in HIF-1α protein levels (57). In addition, inhibitors of topoisomerase I, such as topotecan, an analog of camptothecin, also appear to inhibit HIF-1α protein expression through a mechanism that may depend on a posttranscriptional response by miRNAs (56, 58). HIF-1α mRNA expression and translation are also impacted by PX-478, an inhibitor of HIF-1α deubiquitination processes that results in the proteasome-mediated degradation of HIF-1α (54). As previous studies have shown that mTOR plays a role in HIF-1α translation and activation, mTOR inhibitors such as temsirolimus and metformin would lead to the downregulation of HIF-1α (56). Furthermore, inhibiting HIF-1α binding to HREs on DNA with inhibitors such as doxorubicin prevents the transcription of HIF-1α target genes (59). Evidently, multiple HIF-related treatment methods may be repurposed for use as antiviral therapeutics. However, it is important to note that HIF-1α inhibitors often lack specificity and may perturb separate targets within the host cell and exacerbate infection severity (56).

While many viruses thrive in hypoxia and utilize elevated HIF signaling to their benefit, others are largely impaired by these same processes. As previously described, VSV is a prime example of this phenomenon, as clear cell renal cell carcinoma (RCC) cells lacking VHL, with elevated HIF-2α levels, exhibited greater resistance to VSV infection compared with RCC cells reconstituted with WT VHL (40). While exceedingly rare, infection of normal human tissues by VSV outside of OV therapy is possible and has been reported to cause a variety of symptoms, ranging from a mild flu-like sickness to more severe complications such as viral encephalitis (60). HIF-α stabilizers, such as mimosine, or drugs targeting proteins that regulate HIF-α degradation, such as PHD inhibitors, could be repurposed as antiviral therapeutics against viruses that are impaired by HIF signaling (Figure 3) (9, 61). HIV can also be considered as a virus that is hindered by components of the HIF signaling pathway, but this same pathway can inhibit the reactivation of HIV from latency. This phenomenon interferes with the recognition and targeting of infected cells following viral activation — a strategy utilized for the development of potential therapeutics (62). Therefore, combination therapies that target HIF-2α or enhance the activity of HIF inhibitors may be useful in treating particular HIV strains (30).

Notably, HIF-1α has been shown to decrease the expression of ACE2 in hypoxic cells (50). ACE2 also has roles independent of its involvement as the SARS-CoV-2 receptor. In this capacity, it is involved in antiinflammatory processes and acts in opposition to the RAS (63). Immunopathology has been shown to drive later phases of the disease; here, ACE2 expression may be beneficial to patients (64). Since the SARS-CoV outbreak, there has been increased interest in the involvement of ACE2 and RAS in pathologies such as acute lung injury and ARDS. In animal models, RAS deregulation can further exacerbate acute lung failure as a result of SARS-CoV infection, possibly through the downregulation of ACE2 expression on cells (65). As ACE2 aids in the prevention of lung edema and acute lung failure, its expression may thus be beneficial for patients (65). Given SARS-CoV-2’s similarity to SARS-CoV, a similar process involving a reduction in ACE2 may also be implicated in COVID-19–related multiple organ injuries (63). Recently, the efficacy of corticosteroids in combating COVID-19 disease severity has been widely analyzed (66, 67). In addition to its antiinflammatory properties, dexamethasone has been shown to both inhibit HIF-1α and reduce global expression of ACE2 (68, 69). Recently, it was found that in patients with prolonged symptoms, treatment with dexamethasone led to increased survival rates compared with placebo or standard treatment (70). Therefore, while increased ACE2 expression facilitates initial SARS-CoV-2 infection, its expression following symptom onset could aid in resolving acute lung injury. As side effects of dexamethasone are common, the use of HIF-inhibitors may be of interest as an alternative therapeutic strategy. However, further studies will be required in order to test this hypothesis.

## Conclusion

As the frequency of novel emerging viral infections increases, there is an urgent need to better understand the mechanisms surrounding host-pathogen interactions. Although recent studies have analyzed the role of hypoxia and HIF signaling in host immunity and in various states of infection, its involvement in viral pathogenesis remains largely unclear. Elucidating the function of HIF signaling in either facilitating or inhibiting viral replication could assist in the development of therapeutics. Furthermore, understanding the possible effect that hypoxia can exert on the immune response in both mild and severe infections may allow for more specialized treatment options for patients.

## Author contributions

RH and MH conceptualized, researched, and wrote the manuscript. ESG researched, wrote, and edited the manuscript. EEO supervised ESG, and wrote and edited the manuscript. MO supervised RH and MH, and conceptualized, wrote, and edited the manuscript. Co–first authorship was assigned according to alphabetical order by last name.

## Figures and Tables

**Table 1:**
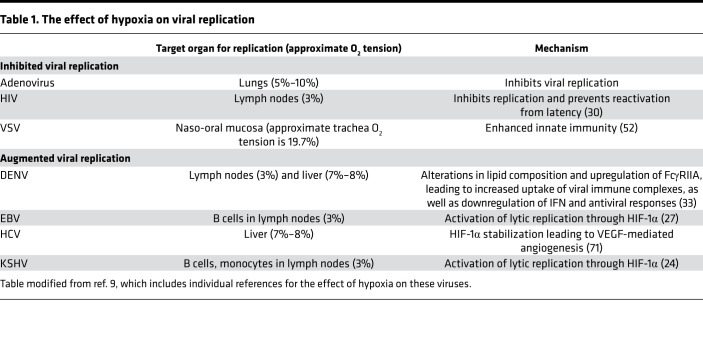
The effect of hypoxia on viral replication

## Version 1 04/08/2021

Electronic publication
